# A Case of Morbid Growth of the Gums

**Published:** 1859-12

**Authors:** J. P. H. Brown

**Affiliations:** Dentist, Atlanta, Ga.


### ARTICLE IV.

*A Case of Morbid Growth of the Gums.* By J. P. H. Brown, *Dentist, Atlanta, Ga.*

There is no disease of the Gums (except a malignant type) that tries more the skill and remedial resources of the Physician or the Dentist, than that form attended with a morbid growth of those parts where the enlargement is often so considerable as to cover entirely the crowns of the teeth. This enlargement has been confounded by several writers with hypertrophy; but it is entirely different. Hypertrophy is not of itself a morbid process, though we frequently find it connected with disease. While, on the other hand, in the morbid growth of the gums, the vital properties of the molecules are altered, and their arrangement seems to be different from what it is in the normal tissue.

This singular affection appears only to occur in persons possessing certain constitutional tendencies, and is always excited by local irritants—by salivary calculus; a crowded, or misplaced condition of the teeth; dead fangs, &c.

The following case came up for treatment during the month of last September. The subject was a gentleman, aged about 27, and possessed a scrofulous diathesis. His gums were very much enlarged in both maxillæ, but more in the upper than in the lower. The molars and bicuspids, on the right side, were nearly covered, rendering the process of mastication very painful. The gums were of a dark, livid color; bled upon the slightest touch; and were attended at times with a prickling or itching sensation. Their edges were thick, hung loose around the sides of the teeth, and discharged a fœtid purulent matter when pressed upon.

During the first sitting, I cut away the greatest portion of this morbid growth with the gum lancet, by making a horizontal incision around the gums down to the necks of the teeth. Nitrate of silver, in stick, was then applied. After giving the patient a solution of the caustic, to be applied to the gums with a camel's-hair pencil twice a day, I sent him to his physician, with the understanding that he was to return to me in the course of a week.

By the way, I may remark that his physician ordered, ℞. Chlorate of Potash, ʒ i., aqua ℥ vi. A table spoonful to be taken three times a day. A bland diet was recommended, and all salted meets were to be eschewed. In all cases of this kind, local treatment alone is not sufficient, and no dentist, but a *machine*, would think of relying upon it. I send many patients, for the good of their *teeth* to the physician, and I know many intelligent physicians who send some of their patients, for the good of their *bodies*, to the dentist.

When I saw the patient again, I found the gums were beginning to assume a healthy condition, but the teeth were badly coated with tartar, which I thoroughly removed. At this sitting I also removed, with a lancet and a tri-angular shaped scaling instrument, all remaining portions of morbid gum, down to the alveolar processes. An astringent gum-wash was now used. In three weeks time, his gums were restored to a sound, healthy condition.

In this case, the tartar which had insinuated itself up under the free margin of the gums, was undoubtedly the exciting cause of the disease, which was favored in its development by the peculiar constitutional tendencies of the patient. In a person of a healthy, vigorous constitution, the same immediate or exciting cause would probably have only manifested itself in a slight inflammation of the gums.

